# Knockout of *dhx38* Causes Inner Ear Developmental Defects in Zebrafish

**DOI:** 10.3390/biomedicines13010020

**Published:** 2024-12-26

**Authors:** Mengmeng Ren, Xiang Chen, Liyan Dai, Jiayi Tu, Hualei Hu, Xiaohan Sun, Jiong Luo, Pei Li, Yiyang Fu, Yuejie Zhu, Weiqiang Sun, Zhaohui Tang, Mugen Liu, Xiang Ren, Qunwei Lu

**Affiliations:** 1 Key Laboratory of Molecular Biophysics of Ministry of Education, College of Life Science and Technology, Center for Human Genome Research, Huazhong University of Science and Technology, 1037 Luoyu Road, Wuhan 430074, China; d202180775@hust.edu.cn (M.R.); 13122500893@163.com (X.C.); d202380948@hust.edu.cn (L.D.); d202080697@hust.edu.cn (H.H.); d202280836@hust.edu.cn (X.S.); workforjiong@163.com (J.L.); lipei814@163.com (P.L.); fuyiyang0501@163.com (Y.F.); zhuyjjane@163.com (Y.Z.); sunweiqiang970216@163.com (W.S.); zh_tang@hust.edu.cn (Z.T.); lium@hust.edu.cn (M.L.); 2 Section of Hematology and Oncology, Department of Medicine, The University of Chicago, Chicago, IL 60637, USA; jiayitu@uchicago.edu

**Keywords:** inner ear development, *dhx38*, zebrafish, cell apoptosis, DNA damage

## Abstract

**Background**: Alternative splicing is essential for the physiological and pathological development of the inner ear. Disruptions in this process can result in both syndromic and non-syndromic forms of hearing loss. DHX38, a DEAH box RNA helicase, is integral to pre-mRNA splicing regulation and plays critical roles in development, cell differentiation, and stem cell maintenance. However, its specific role in inner ear development remains undefined. Here, we utilized a *dhx38* knockout zebrafish model to monitor the ear morphology and elucidate a crucial role for DHX38 in the development of the zebrafish inner ear. **Methods**: Bright-field morphological analysis and in situ hybridization were performed to observe ear morphology changes. Immunofluorescence and semi-quantitative RT-PCR were employed to test apoptotic cells and abnormal splicing. **Results**: The *dhx38^-/-^* mutant zebrafish showed significant inner ear impairments, including decrescent otocysts, absent semicircular canal protrusion, and smaller otoliths. These structural abnormalities were accompanied by substantial DNA damage and p53-dependent apoptosis within the inner ear cells. Alternative splicing analysis showed that genes related to DNA damage repair and inner ear morphogenesis are abnormal in *dhx38* knockout mutants. In summary, we suggest that *dhx38* promotes cell survival during the inner ear development of zebrafish by ensuring the correct splicing of genes related to DNA damage repair.

## 1. Introduction

According to a World Health Organization (WHO) report, over 1.5 billion people are currently affected by hearing loss, and it is projected that approximately 2.5 billion people will be living with some level of hearing loss by the year 2050 [1]. Genetic factors contribute to more than 50% of hearing loss cases [2]. Notably, alternative splicing (AS) defects in the development and function of the inner ear could result in syndromic or non-syndromic hearing loss [3], but the regulation of alternative splicing in the inner ear remains elusive.

Growing evidence shows that alternative splicing is indispensable for the development and physiological function of the inner ear [3]. Nakano et al. reported that the splicing factor Srrm4 is expressed in the sensory hair cells and the spiral ganglion and that its deficiency causes hair cell loss, deafness, and balance defects [4]. Insertional mutation of the splicing factor Sfswap causes vestibular and cochlear defects [5]. Recently, splicing variants of MYO15A were identified in autosomal recessive non-syndromic sensorineural hearing loss [6,7]. The disrupted RNA splicing of *TECTA* and *TMC1* is attributed to autosomal dominant hearing loss [8,9]. Four splicing variants were analyzed in six families with late-onset non-syndromic hearing loss [10]. However, the mechanisms regulating alternative splicing and why deficits of these result in developmental and functional abnormalities of the inner ear still need to be clarified.

The inner ear of zebrafish closely resembles the structure of the human ear. Additionally, the high fecundity, strong conservation, and optical transparency of zebrafish make them valuable tools for research on the development and function of the inner ear of vertebrates [11,12]. The zebrafish genome is highly orthologous to the human genome, facilitating our understanding of vertebrate development and genetic pathology [13]. The mammalian ear comprises the outer, middle, and inner ear, while zebrafish possess only inner ears. Despite the structural diversity across species, the molecular mechanisms underlying inner ear development are largely conserved [14]. Some scholars discovered that the deafness gene *dfna5* regulates the production of hyaluronic acid (HA) in zebrafish; the knockdown of *dfna5* resulted in the disorganization of the developing semicircular canals [15]. Additionally, knockout of the transcription factor *mafba* triggered the activated apoptosis of p53, leading to expanded otocysts and improper formation of otoliths [16]. Busch-Nentwich et al. utilized zebrafish to investigate the mechanisms underlying inner ear hair cell regeneration [17]. Their report demonstrated that the knockdown of *ftr82* led to the loss of hair cells, primarily due to cell apoptosis [18]. THOC1 deficiency results in late-onset, progressive hearing loss through the p53-mediated apoptosis of hair cells in zebrafish [19]. Furthermore, it has been shown that BPA induces otolith malformation by inhibiting otop1 and stm via the activation of the MEK/ERK-EZH2-H3K27me3 signaling pathway in zebrafish [20]. Unfortunately, no zebrafish models of splicing factors in the inner ear have been created, as far as we know.

Ear development originates from ectodermal thickening, known as the otic placode, which is distinguishable from the mid-somite stage on both sides of the hindbrain. In zebrafish, the placode cavitates to form a hollow ball of epithelium, that is, the otic vesicle, from which other structures arise [21,22]. The inner ear of larval zebrafish mainly consists of five sensory patches of epithelium, including two maculae and three cristae. Each of the two maculae is covered by an otolith, a crystalline deposit of calcium carbonate and protein. Morphologically, two otolith organs and three vertical semicircular canals can be observed. The otoliths became distinguishable at 24 h postfertilization (hpf), while the three cristae differentiate slightly later. The semicircular canals are formed by epithelial cells that grow into the middle of the lumen, called protrusions, which are visible at 45 hpf [21]. Subsequently, the protrusions extend inward from the wall of the otic vesicle into the lumen, where they meet and fuse to form the semicircular canal [23]. The morphological irregularity of the developing inner ear often leads to balance and hearing impairments [24].

Dhx38 is an ATP-dependent RNA helicase that was found to be involved in pre-mRNA splicing [25]. It encodes pre-mRNA splicing factor PRP16, which participates in the first and second steps of catalytic pre-mRNA splicing [26]. According to previous publications, DHX38 is the RNA helicase that binds to mitotic noncoding RNA, and its deletion impairs mitotic function and results in an aberrant chromatin arrangement in the mitotic M phase [27]. DHX38 functions as an endogenous inhibitor of the protein phosphatase PP4 by inhibiting the dephosphorylation activity of PP4C/PP4R2 subunits [28]. Prp16 mutations affect a small subset of introns with weak 5′SS-U6 snRNA interactions in fission yeast [29]. A previous study identified a missense mutation in *dhx38* that caused early-onset retinitis pigmentosa with macular coloboma [30]. Mutations in *DHX38* are associated with nonsyndromic autosomal recessive retinitis pigmentosa [31]. During hematopoiesis, *dhx38* plays a crucial role in the maintenance and differentiation of erythro-myeloid progenitors and hematopoietic stem cells through alternative splicing [32]. DHX38/PRP16 may have a role in the carcinogenesis of ovarian clear cell carcinoma (OCCC), indicating its potential as a therapeutic target [33]. We discovered that *dhx38* is expressed in the head, including the region of the inner ear, from the 1-cell stage to hatching: Pec-fin in the zfin database. This expression pattern suggests the potential role of *dhx38* in inner ear development, which is not yet well defined.

In this study, we utilized the *dhx38* knockout zebrafish line to investigate *dhx38*’s role in inner ear development during embryogenesis. The heterozygous individuals exhibited no abnormalities, while the homozygous knockout individuals displayed significant defects in inner ear development. Further research revealed that *dhx38* regulates alternative splicing events and the expression of genes involved in DNA repair and inner ear formation. Excessive splicing errors lead to DNA damage, resulting in numerous apoptosis. This study is the first to elucidate the regulatory roles of *dhx38* in facilitating the alternative splicing of associated genes during inner ear development. Our findings provide novel insights into the development of the inner ear and propose a suitable model to further study the development of inner ear components, such as the semicircular canal, thereby providing tools to test new therapeutic interventions for developmental defects of the inner ear.

## 2. Materials and Methods

### 2.1. Zebrafish Maintenance and Procedure

Zebrafish (Danio rerio) adults were raised and maintained in recirculating water with a constant temperature of 28.5 °C under a 14 h light/10 h dark cycle [34]. We collected and maintained embryos in E3 medium (5 mM NaCl, 0.17 mM KCl, 0.33 mM CaCl_2_, 0.33 mM MgSO_4_). If needed, 0.003% 1-phenyl-2-thiourea (PTU) (Sigma, Shanghai, China) was added to the E3 medium at 12 h after fertilization to prevent the pigmentation of embryos. The hours postfertilization (hpf) and days postfertilization (dpf) were used to record the embryos at different developmental stages. All the zebrafish experiments and maintenance procedures were performed in accordance with guidelines approved by the Experimental Animal Ethics Committee of Huazhong University of Science and Technology.

### 2.2. Whole-Mount In Situ Hybridization

Whole-mount in situ hybridization (WISH) for zebrafish embryos was performed as described previously [16,35]. Zebrafish embryos were collected and fixed with 4% paraformaldehyde (PFA) at 4 °C overnight. Then, they were dehydrated in a gradient of anhydrous methanol (25%, 50%, and 75%) and kept in methanol at −20 °C. The sequences of primers used to compound probes are listed in Supplementary Table S1.

### 2.3. TUNEL Assay Detection

This assay was executed as described previously [36]. The 36 hpf and 48 hpf zebrafish embryos were fixed in 4% paraformaldehyde overnight at 4 °C or at room temperature (RT) for 4 h. If required, the embryos were dehydrated in 100% methanol at −20 °C for at least 30 min or stored at −20 °C for a long time. After gradient rehydration using 75% methanol, 50% methanol, and 25% methanol, the embryos were washed with phosphate-buffered saline (PBS)/Tween. Then, they were digested with proteinase K (20 µg/mL) at RT for 15–20 min and washed once again. The apoptosis cells were labeled with the TUNEL Brightgreen Apoptosis Detection Kit (Vazyme Biotech, Nanjing, China). To better mark the position of the inner ear, the embryos were incubated with DAPI (5 μg/mL), and images were captured using a confocal microscope (FV31S-SW, Olympus, Tokyo, Japan).

### 2.4. Whole-Mount Immunofluorescence Assay

The whole-mount immunofluorescence assay was performed as previously described [16]. To immunostain the whole-mount embryos, they were fixed overnight at 4 °C in 4% PFA in PBS. Then, they were washed with PBST (PBS + 1‰ Triton X-100, Thermo Fisher Scientific, Waltham, MA, USA) three times for 5 min each. Then, the embryos were permeabilized in solution (PBST +1% Triton X-100) to dissolve the otolith for 2–6 h at room temperature until the otolith was dissolved completely. After washing them with PBST for 5 min, the embryos were blocked with 10% goat serum in 0.5% PBST for 2 h. Next, they were incubated with primary antibodies γH2AX (9178s, CST, Danvers, MA, USA; 1:200) at 4 °C overnight. After washing them four times for 2 min each, we treated the embryos with the corresponding fluorescent secondary antibody (Alexa-Fluor 594 -nm secondary antibody, Abcam, Shanghai, China, 1:1000) for 2–4 hpf at room temperature in the dark. The sample images were obtained using a confocal microscope.

### 2.5. Quantitative RT-PCR

The operation method was executed as previously described [16,34]. The heads of the zebrafish embryos were used for total RNA extraction at 36 and 48 hpf. We cut off the tails for genotyping. Approximately 25 heads were dissected and assembled for each specimen to extract RNA using the TRIzol Reagent (Vazyme Biotech, Nanjing, China). We used agarose gel electrophoresis and a microspectrophotometer (K5800 KAIAO, Beijing, China) to demonstrate the quality and concentration of the extracted RNA. Then, we used TransScript All-in-One First-Strand cDNA Synthesis SuperMix (TransGen Biotech, Beijing, China) to reverse-transcribe the RNA into cDNA. Next, qPCR was performed with AceQTM qPCR SYBR Green Master Mix (Vazyme Biotech, Nanjing, China) using QuantStudio™ Design & Analysis Software v1.5.1. (Life Technologies, Carlsbad, CA, USA). The relative differences in the mRNA expression levels of the selected genes were computed using the 2^−ΔΔCt^ method. The gapdh was arranged as an internal control. All the qPCR primers used in this investigation are shown in Supplementary Table S1.

### 2.6. Semi-Quantitative Reverse-Transcription PCR (Semi-RT-PCR) and Splicing Efficiency Analysis

To determine the differential splicing efficiency of homozygotes and their siblings, total RNAs were extracted from the ‘head’ parts. The accurate RNA extraction and cDNA synthesis methods were carried out as previously described. All the primers used in this research are listed in Supplementary Table S1. The PCR products were isolated using electrophoresis on a 2% agarose gel and photographed with XRS^+^ (Bio-Rad, Hercules, CA, USA). Quantification of the DNA lines was performed using ImageJ (v1.8.0). The rate of PSI (percent splicing in), which varies between 0 and 1 for evaluating the proportion of junction reads, was calculated as the percentage of accurate splicing from the total junction reads.

### 2.7. Statistical Analysis

All the experiments were independent and repeated at least three times. The number of samples used in each group is shown in the Materials and Methods Section or the figure legends. Statistical analyses were carried out using GraphPad Prism 6.0 software. Student’s two-tailed *t*-test or one-way ANOVA was used to determine the significant differences in the data. The results are displayed as the mean ± SD. The degrees of significance were set to *p* < 0.05, and *p* > 0.05, *p* < 0.05, *p* < 0.01, *p* < 0.001, and *p* < 0.0001 and labeled as ns, *, **, ***, and ****, respectively.

## 3. Results

### 3.1. Loss of dhx38 Function Results in Inner Ear Defects

**Figure 1:**
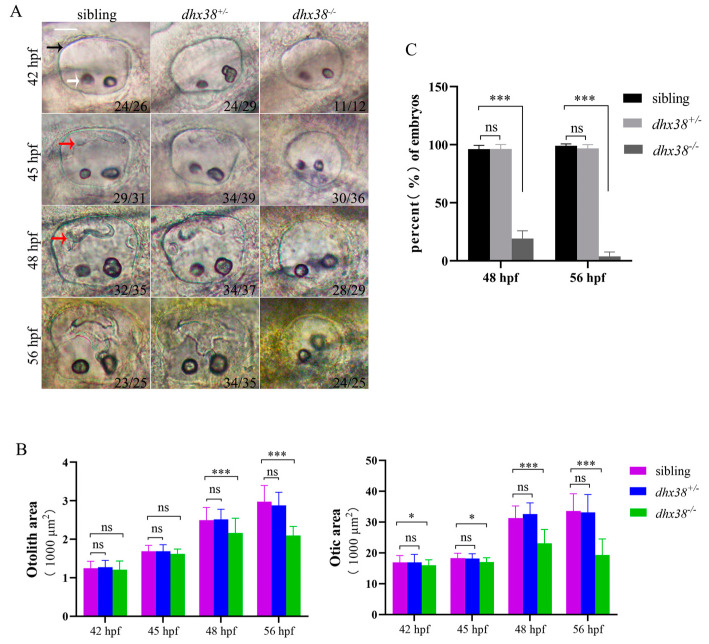
The deletion of *dhx38* caused inner ear development defects: (**A**) The different morphological specificities of the inner ears of different genotypes at distinct developmental stages. Scale bar: 40 µm. The black arrow indicates the otic vesicle, the white arrow indicates the otolith, and the red arrow indicates semicircular canal protrusion. (**B**) Statistical analysis of the otic area and otolith area in the embryos of different genotypes at 42 hpf, 45 hpf, 48 hpf, and 56 hpf. Individual embryos were picked casually from particular types for statistical analysis. The otic and otolith areas were measured using SPOT Advanced software (version 4.6) in the focal plane exhibiting the maximal area [16]. n, the number of checked embryos. n = 40. (**C**) The percentages of embryos that had semicircular canal protrusions in the sibling, the *dhx38^+^*^/*−*^ and *dhx38^−^*^/*−*^ groups at 48 hpf and 56 hpf. The data are presented as the mean ± SD. ns, *p* > 0.05; *, *p* < 0.05; ***, *p* < 0.001.

The establishment of *dhx38* mutant zebrafish has been previously described [32]. We found that the expression pattern of *gata2a*, the homologous gene of *gata2* [37,38], which is expressed in the dorsolateral epithelium of ear vesicles in wild-type mice, differed significantly between the inner ears of wild-type (wt) and *dhx38^−^*^/*−*^ zebrafish (Figure S1A). This indicates that *dhx38* may play a role in the development of zebrafish inner ears. To determine whether *dhx38* is required for inner ear development in zebrafish, we examined the morphological defects in the *dhx38^−^*^/*−*^ homozygous embryos and compared them with those of the sibling embryos. The inner ears of the wt zebrafish included otocysts, otic epithelia, and otoliths at 24 hpf. The otic vesicles of the homozygous mutants were indistinguishable in gross morphology from those of the wild-type and heterozygous siblings at 24 hpf (Figure S2). At the same time, the sizes of the otic vesicles in both the wt and homozygous mutants were similar, with each containing two otoliths. At 36 hpf, there was no obvious difference in the otic vesicle and otolith size or shape between the wt and homozygous individuals (Figure S2). Morphological abnormalities of the inner ear abruptly emerged in the *dhx38^−^*^/*−*^ embryos at 42 hpf (Figure 1A). The *dhx38^−^*^/*−*^ mutants had much smaller otic vesicles, but their otolith areas were comparable to those of the wt (Figure 1B). Besides the otic vesicle defect, there was no protrusion observed at 45 hpf, when the semicircular canal began to form. Until 48 hpf, we could clearly observe semicircular canal protrusions in the wt embryos, but there were still few semicircular canal protrusions in the *dhx38^−^*^/*−*^ homozygous mutants (Figure 1C), and the area of the otic vesicle decreased significantly (Figure 1B). To determine whether the semicircular canal protrusions are delayed development in the *dhx38^−^*^/*−*^ homozygous embryos, we examined the semicircular canal protrusions and otic vesicles at 56 hpf. The semicircular canal protrusions still did not form at 56 hpf (Figure 1C), and the otic vesicles became considerably smaller in the *dhx38^−^*^/*−*^ homozygous embryos (Figure 1B). Notably, the inner ear structures of the *dhx38^−^*^/*−*^ mutants were almost destroyed at 56 hpf. These results support the hypothesis that *dhx38* is critical for inner ear development in zebrafish.

### 3.2. Deficiency of dhx38 Does Not Affect Otic Patterning

The live bright-field images of the siblings and *dhx38^−^*^/*−*^ mutants showed differences at 42 hpf. The early development of the inner ear appeared normal in the *dhx38^−^*^/*−*^ mutants. At 18 hpf, the induction of the otic placode was observed as expected in zebrafish. To understand the molecular function of *dhx38* in otogenesis, we assessed the expression levels of the marker gene involved in the patterning of the inner ear. The expression of the dorsal otic vesicle patterning marker, *dlx3b*, was identical between the sibling and *dhx38*^−/−^ mutant embryos (Figure S1B).

**Figure 2:**
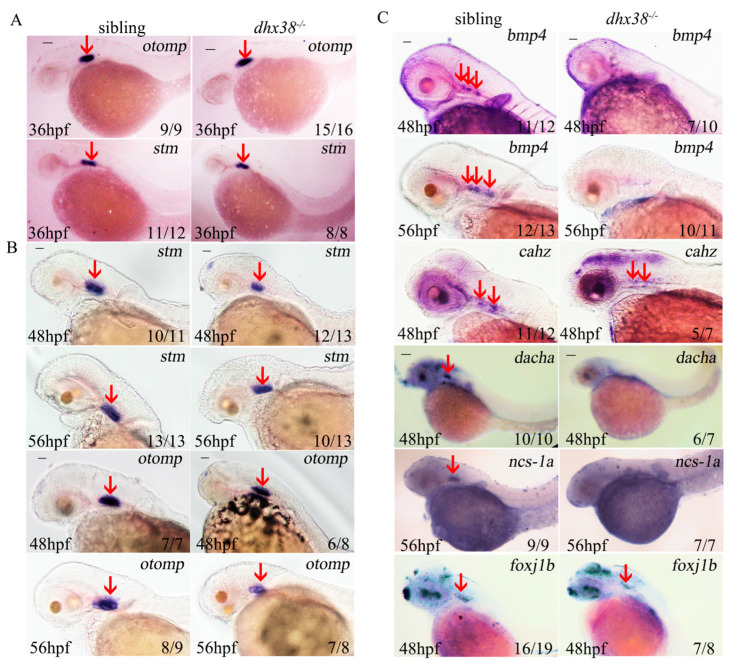
The expressions of some specification-related inner ear markers differed significantly between the sibling and *dhx38^−^*^/*−*^ mutant embryos: (**A**) The expressions of two otic matrix protein markers were normal at 36 hpf in the sibling and *dhx38^−^*^/*−*^ mutant embryos. Scale bar: 100 µm. (**B**) Two otic matrix protein markers displayed slight declines at 48 hpf and 56 hpf. Scale bar: 40 µm. (**C**) The in situ staining of markers for semicircular canal sensory cristae (*bmp4*), and saccular and utricular maculae marker (*cahz*), respectively. Scale bar: 40 µm. The epithelial pillar of the semicircular canal (*ncs-1a*), semicircular canal projections (*dacha*), and cilia (*foxj1b*). Scale bar: 100 µm. The red arrows indicate signal spots.

To determine whether the formation of each part of the inner ear was abnormal in the mutants, we further examined various markers using in situ hybridization assays. The matrix protein genes *otomp* and *starmaker* (*stm*) are essential for the development of otoliths [39,40]. Consistent with the results of the bright-field images, the expression patterns of the two otolith markers *otomp* and *stm* showed no significant differences between the sibling and *dhx38^-/-^* mutant embryos at 36 hpf (Figure 2A). However, the signals and distributions of these markers were significantly reduced in the mutants compared to the siblings at both 48 hpf and 56 hpf (Figure 2B). The saccular and utricular maculae marker, *cahz*, exhibited a slight reduction at 48 hpf (Figure 2C). Foxj1b, which is necessary for the differentiation of motor cilia and intense cilia [41], showed a marginal decrease in signaling (Figure 2C). The semicircular canal sensory cristae marker, *bmp4*, was expressed in the otic region of the wild-type embryos but was nearly absent in the *dhx38^−^*^/*−*^ mutant embryos at 48 hpf and 56 hpf (Figure 2C). *DachA* is primarily expressed primarily in non-sensory regions and marked the anterior and posterior semicircular canal projections at 48 hpf, when the canal began to extend [21]. *Ncs-1a*, which encodes neuronal calcium sensor-1a is necessary for semicircular canal formation [42]. It marks the epithelial pillar of the semicircular canal, which was expressed in the ear epithelium and budding epithelium of the wild-type embryos. Each of these markers was absent in the *dhx38^−^*^/*−*^ homozygotes (Figure 2C). These data suggest that the deletion of *dhx38* had no effect on the otic vesicle patterning during early development prior to 36 hpf, but impaired later development. Combined with the above results, the loss of *dhx38* affected the development of ear vesicles and caused a deficiency of the semicircular canal and otolith phenotype.

### 3.3. Apoptosis Is Increased in dhx38^−/−^ Embryos

The results indicate that the deletion of *dhx38* led to defects in the inner ear epithelium, resulting in the failure of the semicircular canal protrusions. Previous studies have suggested that the semicircular canal protrusions in zebrafish originate from the ear vesicle epithelium [22,43]. Given the significant reduction in the area of the otic vesicle at 42 hpf and the absence of semicircular canal protrusions in the *dhx38^−^*^/*−*^ homozygous inner ear till 56 hpf, along with the progressive impairment of inner ear development, we suspect that apoptosis and proliferation may be abnormal in *dhx38^−^*^/*−*^ mutants.

**Figure 3:**
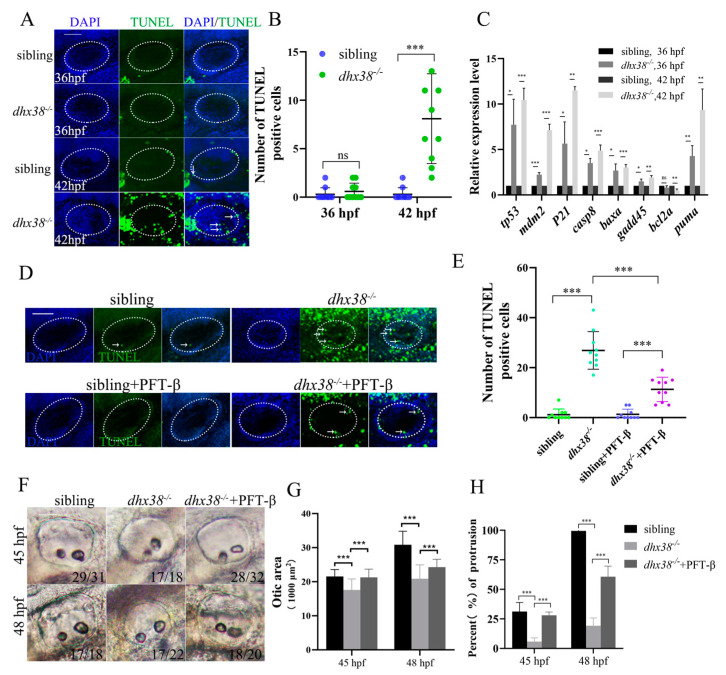
Apoptosis was increased in the inner ear epithelium cells of *dhx38^−^*^/*−*^ zebrafish: (**A**) TUNEL staining in the inner ears of *dhx38* mutants at 36 hpf and 42 hpf. The white arrows indicate the signal spots.The n = 10 for each panel. Scale bar: 50 µm. (**B**) Quantitative analysis of apoptotic cells of the inner ear (the white, dotted, oval area) region between sibling and *dhx38^−^*^/*−*^ mutants. (**C**) The expression level of p53 pathway genes in sibling and *dhx38^−^*^/*−*^ mutants at 36 hpf and 42 hpf using qPCR. (**D**) TUNEL staining indicated that cell apoptosis was inhibited in the *dhx38* zebrafish mutant embryos by inhibiting p53. The white arrows indicate the signal spots. Scale bar: 50 µm. (**E**) Quantitative analysis of TUNEL-positive cells in the inner ear (the white, dotted, oval area) region of siblings, *dhx38^−^*^/*−*^ homozygous embryos, and embryos with p53 inhibitor. (**F**) The inner ear morphology of different genotypes at 45 hpf and 48 hpf. (**G**) The statistical analysis of the otic lumen area at 45 hpf and 48 hpf. (**H**) The statistical proportion of protrusions at 45 hpf and 48 hpf. n = 30. The data are the mean ± SD. ns, *p* > 0.05; *, *p* < 0.05; **, *p* < 0.01; ***, *p* < 0.001.

Given that a previous study demonstrated that localized cell proliferation is not necessary for budding [44], we detected and measured the apoptosis of inner ear epithelial cells in *dhx38^−^*^/*−*^ homozygous embryos using TUNEL assays. The *dhx38^−^*^/*−*^ homozygous embryos exhibited few apoptotic signals at 36 hpf (Figure 3A,B); however, there was a significant increase in apoptotic cell signals in the inner ear region of *dhx38^−^*^/*−*^ homozygous embryos compared to those in the siblings at 42 hpf (Figure 3A,B). At 48 hpf, we identified several apoptotic signals in the *dhx38^−^*^/*−*^ mutants (Figure S3A,B). Increased apoptosis may cause the defects in zebrafish inner ear cells. In summary, *dhx38* is required for the survival of epithelium cells, which supports the normal development of semicircular canal protrusions in zebrafish inner ears.

### 3.4. Deprivation of dhx38 Activates the p53 Apoptosis Pathway

Our preliminary results suggest that the deletion of *dhx38* triggers apoptosis. Previous studies have reported that the activation of the p53 pathway is one of the most widely accepted apoptosis-inducing factors [45,46,47]. Therefore, we conducted experiments to detect the expression levels of several apoptosis-related genes, including *p53*, *caspase8*, *baxa*, and target genes in the *dhx38^−^*^/*−*^ mutants and sibling embryos at 36 hpf and 42 hpf. The quantitative PCR results indicate that *p53* and its target genes, *mdm2*, *p21*, and *puma*, along with apoptosis-related genes *caspase8*, *baxa*, and *gadd45*, were significantly upregulated in the *dhx38^−^*^/*−*^ mutants at 36 hpf (Figure 3C). Similarly, these genes also exhibited a marked increase at 42 hpf (Figure 3C) and showed a slight upregulation at 48 hpf (Figure S3C), although *gadd45* showed downregulation. It is possible that these genes need to express more at 36 hpf to induce apoptosis later at 42 hpf. As apoptosis occurred in many cells, the expression levels began to decrease. Additionally, the expressions of two typical inhibitors of *p53*, *mdm4,* and *bcl2a*, decreased significantly. In summary, these results suggest that the activation of the p53 pathway is one of the reasons for cell apoptosis.

### 3.5. Inhibiting p53 Could Partially Restore Inner Ear Defects

To determine whether p53 activity contributes to inner ear abnormalities, we rescued the embryos using a p53 inhibitor. We collected embryos and detected their apoptosis using TUNEL assays after incubation with the p53 inhibitor PFT-β at 48 hpf. As expected, apoptosis signaling was inhibited, and the number of TUNEL-positive cells was effectively reduced in the *dhx38^−^*^/*−*^ embryos incubated with the p53 inhibitor PFT-β (Figure 3D,E). We then investigated whether the morphology of the inner ear had improved. We observed the morphology of the zebrafish inner ear under bright-field microscopy at 45 hpf and 48 hpf. We found that the otic vesicle areas were partially rescued at both 45 hpf and 48 hpf when p53 was inhibited in the *dhx38^−^*^/*−*^ homozygous embryos (Figure 3F). At 48 hpf, surprisingly, we also observed some protrusions in the *dhx38* mutant embryos treated with the inhibitor. The results of the statistical analysis of the ear vesicle area are presented in Figure 3G. According to these results, the proportion of protrusions increased from approximately 12% to 60% (Figure 3H). These results suggest that the activated *p53* pathway triggered cell apoptosis in the inner ears of the *dhx38^−^*^/*−*^ mutant embryos and it was also responsible for the improper development of protrusions and the defects in inner ear development.

### 3.6. Accumulation of DNA Damage in Inner Ears of dhx38^−/−^ Mutant Embryos

**Figure 4:**
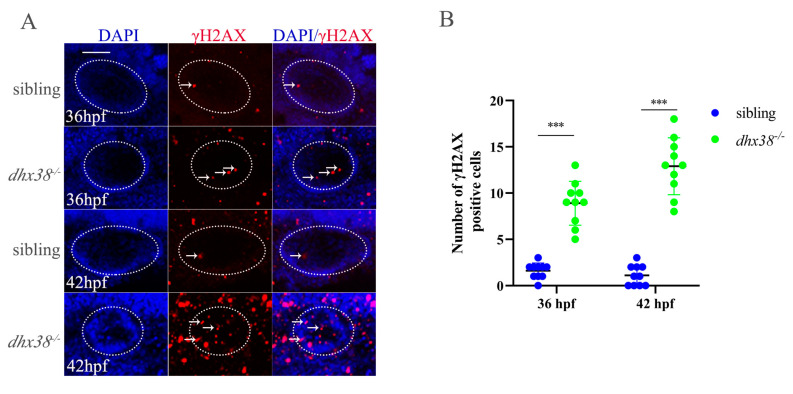
Analysis of the accumulation of DNA damage in inner ears of *dhx38^−^*^/*−*^ mutants: (**A**) Whole-mount immunofluorescence analysis using the anti-γH2AX antibody in inner ear regions of siblings and *dhx38^−^*^/*−*^ at 36 hpf and 42 hpf. The white arrows indicate the signal spots. n = 10 for each panel. Scale bar: 50 µm. (**B**) Quantitative analysis of the γH2AX-positive cells exhibited in the inner ear (the white, dotted, oval area) region in (**A**). The data are presented as the mean ± SD. ***, *p* < 0.001.

The apoptotic cells in the inner ears of *dhx38^−^*^/*−*^ mutant embryos resulted from the activation of the *p53* pathway. Several studies have demonstrated that removing certain splicing factors leads to severe DNA damage both in vivo and in vitro [34,48,49,50]. We speculated that DNA damage may occur in *dhx38^−^*^/*−*^ mutants, which could explain the cause of apoptosis. To prove this hypothesis, we carried out immunofluorescence experiments to identify whether DNA damage existed in the inner ears of *dhx38^−^*^/*−*^ homozygous embryos using γH2AX, a sensitive marker of DNA damage at 36 hpf and 42 hpf (Figure 4A). Compared to the siblings, more γH2AX stained cells were observed in the inner ears of the *dhx38* homozygous embryos at both 36 hpf and 42 hpf. Accordingly, the number of γH2AX-positive cells in the inner ears of the *dhx38^−^*^/*−*^ embryos increased dramatically at 42 hpf compared to those in the sibling embryos (Figure 4B). Additionally, the number of the γH2AX-labeled cells in the inner ears of the *dhx38^−^*^/*−*^ embryos at 42 hpf was more than that at 36 hpf, possibly due to the accumulation of γH2AX-positive cells with the decrease of *dhx38*. There was also more DNA damage in the *dhx38^−^*^/*−*^ mutant embryos at 48 hpf (Figure S3D,E). These results suggest that *dhx38* is essential for preventing DNA damage in the inner ears of zebrafish.

### 3.7. Defects in dhx38 Lead to Abnormal Splicing of Genes in the Inner Ear Involved in DNA Repair

**Figure 5:**
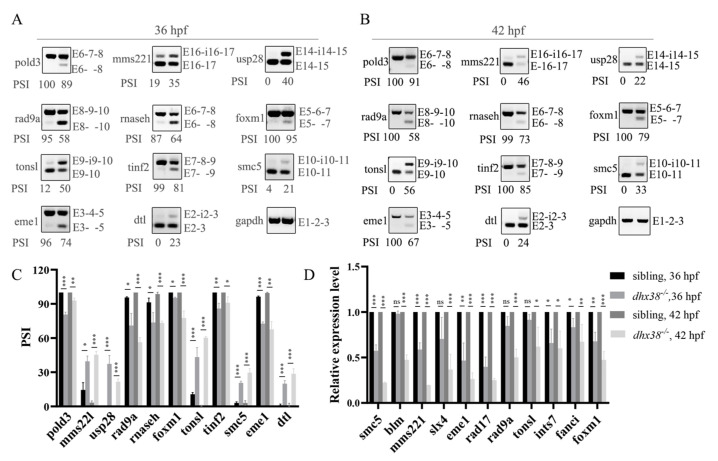
Dhx38 regulates the alternative splicing of some genes involved in DNA damage repair: (**A**) Increased rate of transcripts with IR or ES among DNA repair in inner ears of *dhx38* mutant embryos, as detected by PCR at 36 hpf. (**B**) The alternative splicing of these genes at 42 hpf. (**C**) The statistical data are presented as the mean ± SD of the PSI values at 36 hpf and 42 hpf from three biological replicates. PSI, percent splicing in. (**D**) The mRNA expression levels of some genes in inner ears of sibling and *dhx38^−^*^/−^ mutant embryos with DNA repair, as detected using RT-PCR. The data are presented as the mean ± SD. ns, *p* > 0.05; *, *p* < 0.05; **, *p* < 0.01; ***, *p* < 0.001.

To further explore the potential mechanism by which the deletion of *dhx38* affects DNA damage, we referenced transcriptome data from a previous study and identified genes associated with DNA damage and repair [32]. The data indicated that several genes exhibited abnormal splicing. Therefore, we performed semi-RT-PCR to verify whether aberrant splicing events occurred in the inner ear. The results of the semi-RT-PCR analysis show that several genes underwent aberrant splicing, including exon-skipping (ES) and intron-retention (IR) at 36 hpf and 42 hpf (Figure 5A,B). The PSI values were measured in the sibling and *dhx38* mutant embryos (Figure 5C) and showed that the splicing of these events was severely suppressed in the latter. We then investigated whether the mRNA expression levels were normal with RT-qPCR. The results showed that the mRNA levels of certain genes involved in DNA repair were significantly downregulated (Figure 5D). Additionally, we detected aberrant splicing events at 48 hpf (Figure S4A,B), and the mRNA levels of these genes were also significantly downregulated (Figure S4C). Together, these data suggest that the deletion of the splicing factor *dhx38* primarily affected the alternative splicing of specific genes involved in DNA repair during the early embryonic stage, indicating that the development of the inner ear of zebrafish is sensitive to *dhx38* deficiency.

### 3.8. Dhx38 Knockout Results in Aberrant Splicing of Some Genes Involved in the Formation of the Inner Ear

**Figure 6:**
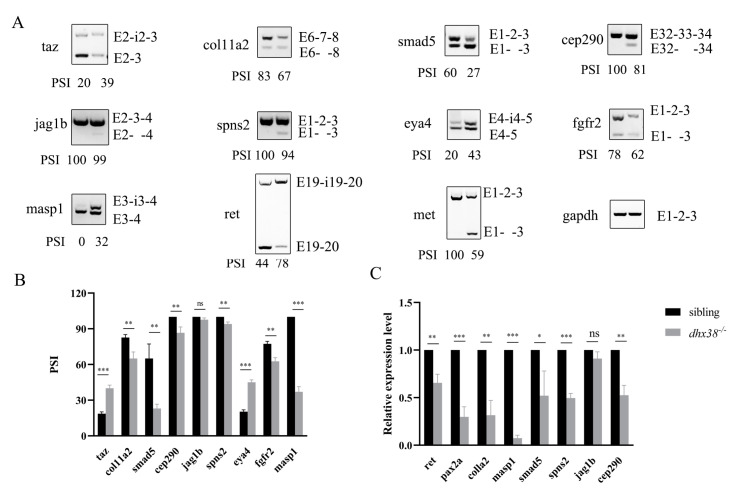
The abnormal splicing of genes involved inner ear development at 42 hpf: (**A**) The occurrence of an abnormal splicing event with IR and/or ES among genes associated with the development of the inner ear. (**B**) The statistical data of the PSI values at 42 hpf from three biological replicates. PSI, percent splicing in. (**C**) The downregulation of some genes with aberrant splicing in *dhx38* mutants was detected using qRT-PCR at 42 hpf. The data are presented as the mean ± SD. ns, *p* > 0.05; *, *p* < 0.05; **, *p* < 0.01; ***, *p* < 0.001.

To investigate the comprehensive effects of *dhx38* knockout on inner ear development in zebrafish, we enriched genes related to inner ear development and organogenesis from transcriptome data [32]. Additionally, we found that these genes may lead to differential splicing (DS) events in vivo. We confirmed this with semi-RT-PCR at 42 hpf. The results showed that *dhx38* deletion significantly affected the alternative splicing events of some genes, several of which resulted in ES, and some of them caused IR (Figure 6A,B). These splicing defects may trigger nonsense-mediated decay (NMD), thereby disrupting proper gene expression. Splicing defects may generate abnormal transcripts, which can reduce the expression levels of normal transcripts and disrupt proper gene expression. The mRNA expression levels were analyzed using RT-qPCR, revealing that the most functional transcripts were downregulated (Figure 6C). The alternative splicing events and the mRNA expression levels at 48 hpf are shown in Figure S5. Eya4 mutation resulted in smaller ear vesicles with short, malformed, and broken epithelial protrusions, leading to distorted semicircular canals [51]. Cilia gene *cep290*, mutations of which are involved in human ciliopathies, regulates CaCO_3_ crystallization and functions in otolith formation [52]. Jag1b is required for the cell survival of the posterior crista, and marks the prosensory and sensory cristae during inner ear development in zebrafish [53]. These genes are indispensable for the development of the inner ear. However, they exhibited unusual splicing and decreased expression in *dhx38^−^*^/*−*^ homozygous embryos. These factors may contribute to the disruption of inner ear development in *dhx38* knockout zebrafish.

To further identify the potential mechanisms underlying undeveloped semicircular canal protrusions, we analyzed several genes that are crucial for semicircular canal development, whose deficiency has been shown to result in the absence of semicircular canal protrusions [15,42,54]. The results demonstrate that they have obviously reduced expression in *dhx38^−^*^/*−*^ mutant embryos (Figure S6). Together, these data indicate that the knockout of *dhx38* could impair the expressions of specific genes involved in protrusions, thereby disrupting inner ear development.

## 4. Discussion

Dhx38 is a splicing factor that is ubiquitously expressed in various organs in zebrafish embryos. Our previous studies have demonstrated its role in retinal and hematopoietic stem progenitor cells [32,55]. The present study further elucidates its involvement in inner ear development. The deletion of *dhx38* results in severe defects, including smaller otocysts, smaller otoliths, and undeveloped semicircular canal projections (Figure 1). Additionally, DNA damage occurs, and *p53* is upregulated, which leads to apoptosis in the inner ear. These inner ear defects can be partially mitigated through p53 inhibition. Subsequent research suggested that *dhx38* plays an important role in the DNA damage response by regulating alternative splicing and the expressions of related genes involved in DNA damage and repair. On the other hand, *dhx38* is required for normal inner ear development by regulating the alternative splicing of several genes involved in this process. This study provides new insights into the role of *dhx38* in inner ear development.

Splicing factor dysfunction consistently leads to abnormal apoptosis and the accumulation of DNA damage, phenomena also observed evidently in inner cells following the knockout of *dhx38*. In ovarian clear cell carcinoma cells, the knockdown of *dhx38* induced apoptosis through p53 [33]. Consistent with this, we found that *dhx38* deletion resulted in severe cell apoptosis in the inner ears of zebrafish through the upregulation of *p53*. Many reports have demonstrated that splicing factor deficiency can cause severe DNA damage [48,50]. Our previous research suggested that the loss of *dhx38* triggers R-loop-dependent DNA damage [55]. Here, significantly more γH2AX-positive cells were detected in the inner ears of *dhx38^−^*^/*−*^ zebrafish (Figure 4), indicating the accumulation of DNA damage. We also detected some genes, such as *rad9a*, which is a key factor in DNA damage response pathways, displayed splicing errors and reduced expression. Reports have demonstrated that Rad9a not only is involved in different mitotic DNA repair processes, recruited by both ATM and ATR signaling pathways, but also has a vital role in maintaining the stability of the genome [56,57]. Hopkins KM found that disrupting rad9a caused lethality in mouse embryos [58]. MMS22L has been suggested to act as a recombination mediator and is necessary for appropriate RAD51 assembly at DNA damage spots in vivo [59,60]. Wojciech Piwko showed that MMS22L–TONSL stimulates DNA recombination directly upon replication stress, and the heterodimer also acts as a recombination regulator at stalled replication forks [60]. In our investigation, some genes involved in genome stability, such as *rad9a*, *mms22l*, and *tonsl*, showed false splicing and decreased mRNA expression (Figure 5). We speculate that the aberrant splicing of these genes in the inner ear may contribute to DNA damage. This is further exacerbated by the decreased DNA repair capacity, causing genomic instability.

The proper splicing of various genes is essential for inner ear development. However, it remains unclear which genes involved in inner ear development are regulated by *dhx38*. Eya4 is expressed in the otic vesicle and neuromast sensory organs, and its loss leads to smaller and misshapen otic vesicles, fewer hair cell numbers, and disrupted sensory responses [51]. Interestingly, mutations in the human EYA4 were found to cause late-onset hearing impairment at the DFNA10 locus [61]. Masp1 has been reported to encode mannan-binding lectin serine protease 1, and its mutations are linked to a human malformation syndrome [62,63]. The col11a2 mutation is associated with non-syndromic hearing loss (DFNA13), as well as autosomal recessive non-syndromic hearing loss DFNB53 [64,65]. There is an association between vertigo in humans and a missense mutation in OTOG [66]. The mis-splicing of *otog* in this study may lead to the loss of existing functions or the gain of new functions, either of which may be deleterious or pathogenic. These findings suggest that the deficiency of *dhx38* results in the abnormal splicing of several pathogenic genes, highlighting the potential for inner ear diseases in *dhx38* knockout zebrafish. Previous studies have suggested that *dhx38* is associated with non-syndromic retinitis pigmentosa (RP) [31,67]. However, since hearing abnormalities are common in syndromic forms of RP, our results suggest that the RP in some DHX38-mutation patients may represent a syndromic form, which may also have inner ear disorders. Although we have focused on inner ear defects, other tissues, and organs, such as the nervous system and heart, may also be affected in patients.

Although we have gained valuable insights into the cellular mechanisms of the inner ear, several questions remain for future research. The inner ear comprises various cell types, including hair cells, supporting cells, sensory epithelium, and non-sensory epithelium. The impact of DNA damage and apoptosis on these specific cell types varies depending on the developmental stage. We speculate that DNA damage and apoptosis primarily occur in non-sensory epithelial cells, as indicated by the initial observation of a smaller ear vesicle area. However, it is possible that these processes also affect other cell types. Therefore, further research is essential to accurately identify the specific cell types involved in *dhx38* knockout zebrafish. Additionally, the early mortality of *dhx38* knockout zebrafish before reaching maturity limits our ability to assess their auditory physiology and vestibular function.

In summary, we discovered development impairment in the inner ear of the *dhx38* knockout zebrafish line for the first time and revealed an unexpected role of *dhx38* in inhibiting DNA damage and maintaining cell survival in the inner ear by modulating the pre-mRNA splicing of genes involved in DNA repair during zebrafish development. This study can help broaden our understanding of the biological functions of *dhx38* in inner ear development, which could provide valuable guidance for the early diagnosis, screening, and prevention of congenital deafness and ear disease and offer tools for testing new therapeutic interventions for developmental defects of the inner ear.

## Data Availability

The data presented in this study are available on request from the corresponding author.

## Supplementary Materials

The following supporting information can be downloaded at https://www.mdpi.com/article/10.3390/biomedicines13010020/s1: Figure S1: The expressions of *gata2* and *dlx3b* during the development of the inner ear of zebrafish. Figure S2: The normal developmental morphology of the inner ear in the early stage. Figure S3: TUNEL staining and DNA damage in the inner ear of zebrafish in sibling and *dhx38* mutants at 48 hpf. Figure S4. The alternative splicing of some genes involved in DNA damage repair at 48 hpf. Figure S5. The abnormal splicing of genes involved during inner ear development at 48 hpf. Figure S6. Quantitative RT-PCR analysis of some genes that have been reported to be extremely important for the development of the semicircular canal at 42 hpf and 48 hpf.; Table S1. Primers used to identify genotypes and synthesize WISH probes; qRT-PCR and semi-RT-PCR.
