# Supplementary material for: Knockout of dhx38 Causes Inner Ear Developmental Defects in Zebrafish
Source: Biomedicines. 2024 Dec 26;13(1):20. doi: 10.3390/biomedicines13010020 (PMC11760894; doi:10.3390/biomedicines13010020)
Supplement: Supplementary file 1 [file biomedicines-13-00020-s001.zip › biomedicines-3328041-supplementary.pdf]

Supplementary Materials

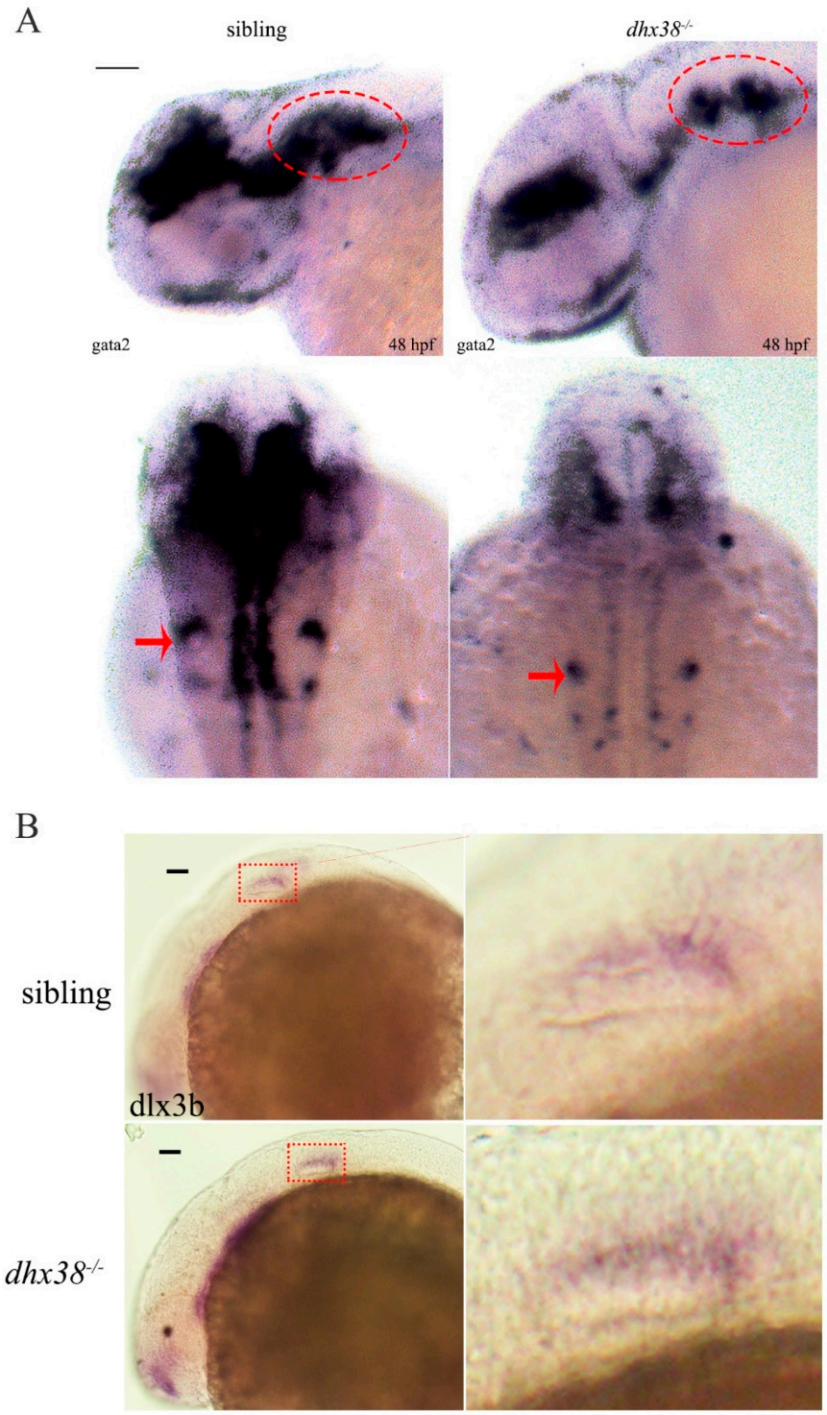

Figure S1: The expressions of *gata2* and *dlx3b* during the development of the inner ear of zebrafish. (A) The inner-ear region are shown by red, dotted, circle, and the red arrow indicate the inner ear(view from backside). Scale bar: 100 μm. (B) Early patterning of the otic vesicle appears normal in *dhx38*$^{-/-}$ mutants. Whole-mount in situ hybridization showed that the expression of early patterning marker in the otic vesicle is indistinguishable between siblings and *dhx38*$^{-/-}$ mutants. Normal expression is seen for *dlx3b* dorsally at 24 hpf. Scale bar: 40 μm. The inner ear is pointed by the red, dotted rectangle.

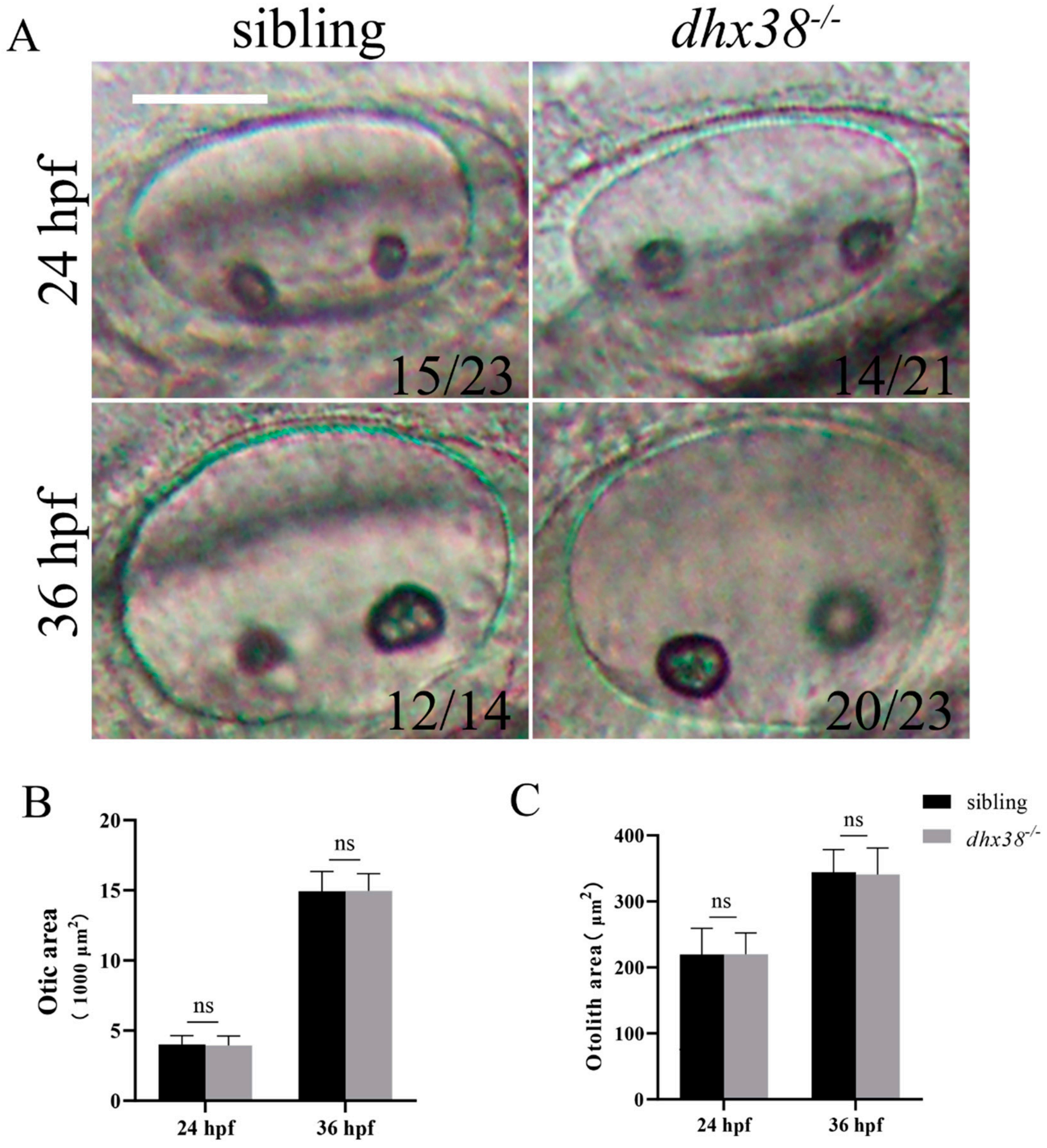

Figure S2: The normal developmental morphology of the inner ear in the early stage. (A) Morphological phenotypes of inner ear in sibling and *dhx38*$^{-/-}$ homozygous embryos at 24 hpf and 36 hpf. Scale bars: 40 μm. (B) and (C) Statistical analysis of the otic lumen area and otolith area in different types of embryos. Individuals of sibling and homozygous embryos were randomly picked for statistical analysis of otic lumen and otolith areas at 24 hpf and 36 hpf. n=20. Data are represented as mean ± SD; ns, $p > 0.05$; **, $p < 0.01$; ***, $p < 0.001$.

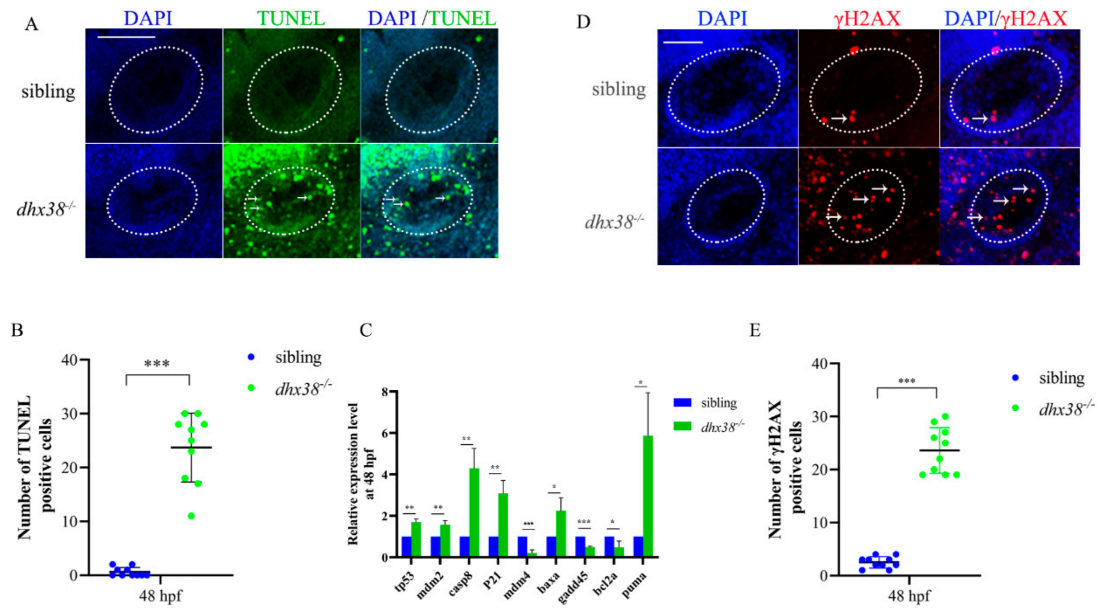

Figure S3: TUNEL staining and DNA damage in the inner ear of zebrafish in sibling and *dhx38* mutants at 48 hpf. (A) There has lots of apoptotic signals in inner ear of *dhx38* mutants. The n = 10 for each panel. Scale bars: 50 µm. (B) The quantitative analysis of apoptotic cells of the inner ear (the white, dotted, oval area) region between sibling and *dhx38*$^{-/-}$ mutants. The signal spots were pointed by white arrows. (C) The p53 pathway was activated in inner ear of zebrafish embryos. The expression level of p53 pathway genes in sibling and dhx38-/- mutants at 48 hpf by qPCR. (D) Whole-mount immunofluorescence analysis using the anti-γH2AX antibody in siblings and *dhx38*$^{-/-}$ in inner ear regions. The white arrows display the signal spots. The n = 10 for each panel. Scale bars: 50 µm. (E) Quantitative analysis of the γH2AX positive cells exhibited in inner ear (the white, dotted, oval area) region in (D). Data are mean ± SD; ns, $p > 0.05$; *, $p < 0.05$; **, $p < 0.01$; ***, $p < 0.001$ compared to the sibling control.

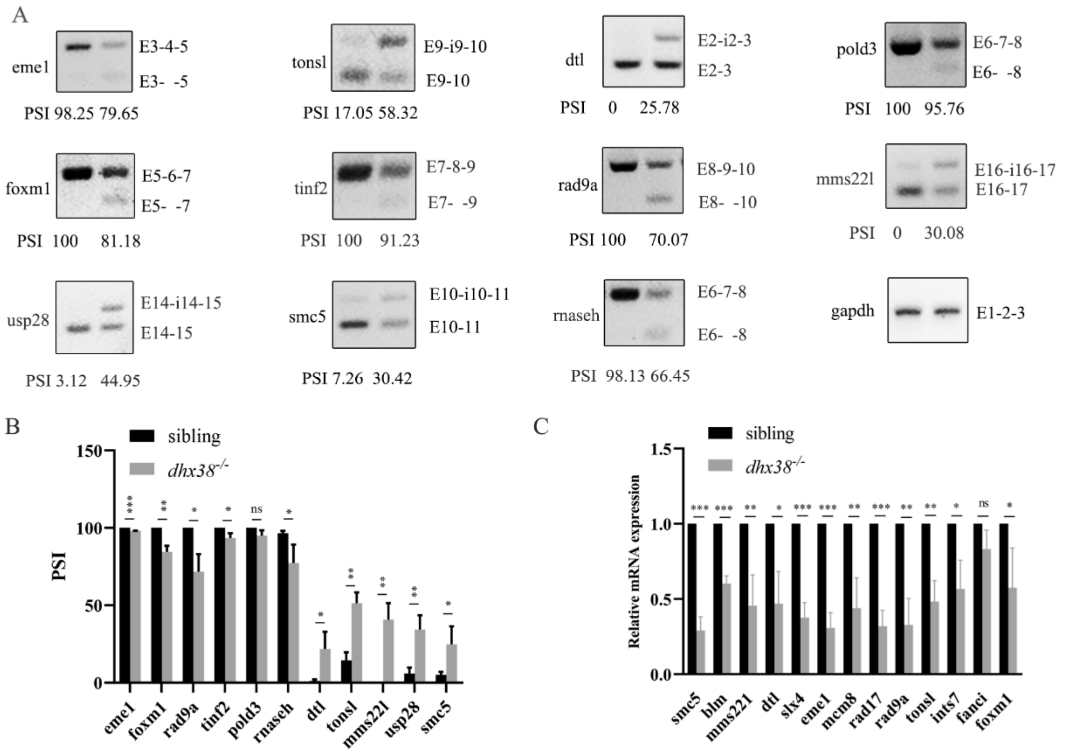

Figure S4. Quantitative RT-PCR analysis of some genes that have been reported to be extremely important for the development of the semicircular canal at 42 hpf and 48 hpf. (A) Increased rate of aberrant splicing events in inner ear of *dhx38* mutant embryos, as detected by semi-RT-PCR. (B) Statistical analysis presented as the mean ± SD of PSI values at 48 hpf. PSI, percent splicing in. (C) The mRNA expression levels of some genes among DNA repair in inner ear of sibling and *dhx38*$^{-/-}$ mutant embryos as detected by RT-PCR. Data are represented as mean ± SD; ns, $p > 0.05$; *, $p < 0.05$; **, $p < 0.01$; ***, $p < 0.001$.

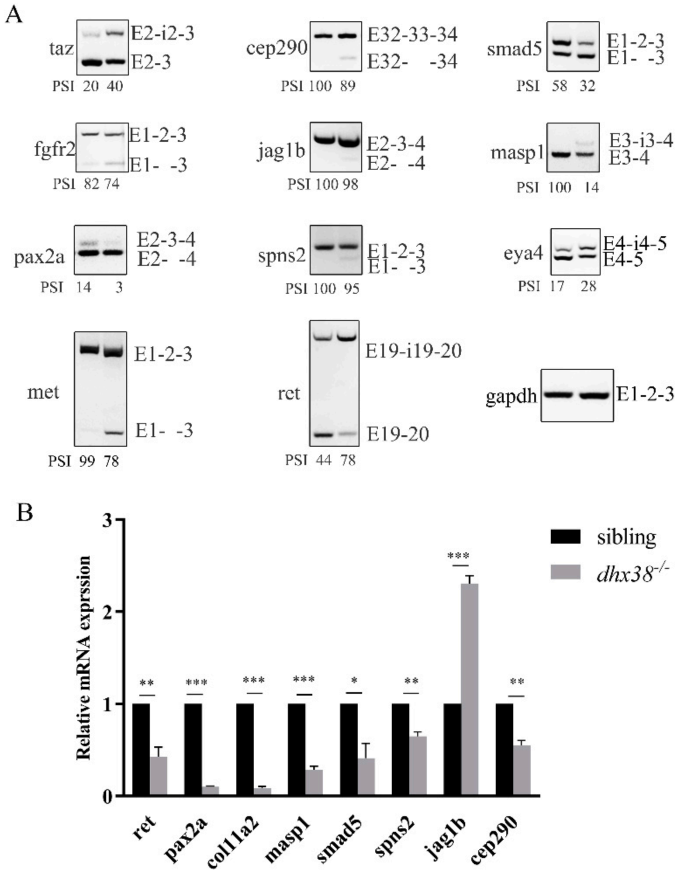

Figure S5. The abnormal splicing of genes involved during inner ear development at 48 hpf. (A) Occurrence of abnormal splicing event with IR and/or ES among genes associated with the development of inner ear. (B) The Quantitative RT-PCR analysis of some genes have been reported to cause inner ear defects when they are defective in sibling and *dhx38*$^{-/-}$ mutant embryos at 48 hpf. Data are represented as mean ± SD; ns, $p > 0.05$; *, $p < 0.05$; **, $p < 0.01$; ***, $p < 0.001$.

A

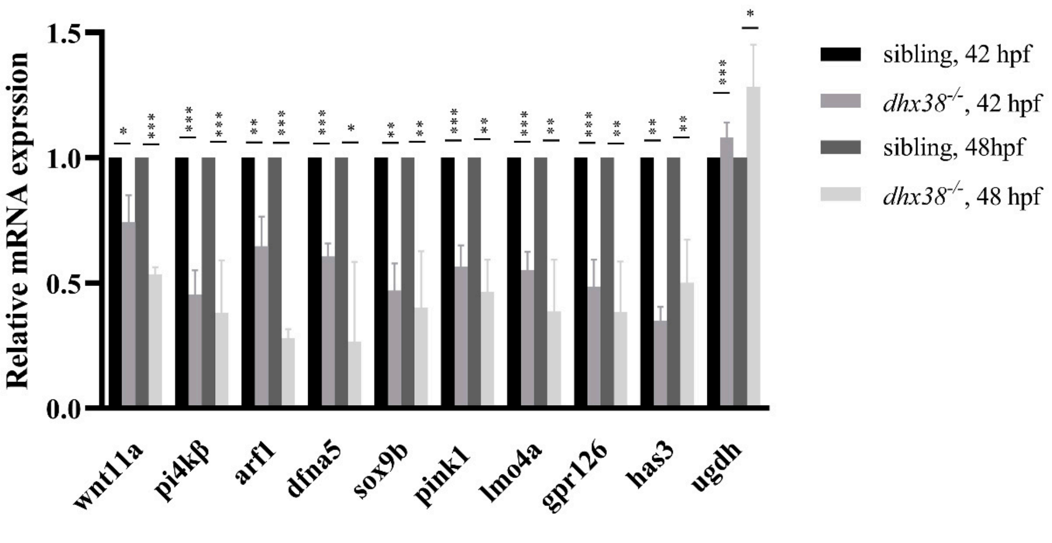

Figure S6. The Quantitative RT-PCR analysis of some genes that have been reported they are extremely important for the development of semicircular canal at 42 hpf and 48 hpf. Data are represented as mean ± SD; ns, $p > 0.05$; *, $p < 0.05$; **, $p < 0.01$; ***, $p < 0.001$ compared to the sibling control.

Table S1. Primers used to identify genotypes and synthesize WISH probes; qRT-PCR and semi-RT-PCR.

PCR

Terms	Primer names	Forward	Reverse
WISH probes	otomp	CACCCAGGGATGAGGACTA	GCACATCAGGCTGCCCATA
	stm	CTGACAGTGCATCGGTAGAGG	GCATAGAAGTTTTCCTGCCATC
	bmp4	CCTTGCTGTGGAGGTTGTA	CGTGATTGGTGGAGTTGAG
	cahz	GAAGTATGACCCAGCCACC	GTCTGTCTGCCCTTTGATT
	foxj1b	GAATGCCTGCCACAAACTT	TGAGAACATCCCCTCGTAA
	dacha	GTGCTGAAGAGCAGTCACCT	TTCTGTCCCTTTGTGTAGCGA
	ncs1a	AGAGGTGCGTTGTGGGAAAT	AAGACTGCCTGTGGGTCAAG
RT-PCR	p53	GGAGGTCTTTTGAGGTGCGT	AGCTGAAGAAGATTTACGTTTGGT
	mdm2	AAGCAGTGATCCTGAGAGTTC	ATCCGAAGACTCGCTGTTC
	mdm4	ATTCGGATGCGGCAAAGA	CCCACTGGACACCTGACC
	caspase8	GGCACTGATATGGACAAAGATAGA	GCATCCGGCAAAAGGCAAAG
	bcl2a	CGAGTTTGGTGGGACCATGT	CGTACATCTCCACGAAGGCA
	puma	TGGAAAGCAGAGTGGACGAA	GATGGCAGGGCTGGATGA
	baxa	GCACTTCTCAACAACTTTGTGTATG	CGCATTTCCATCCAGCTCATC
	p21	TCAGTCATGACAGCTCAGAGGCGC<br>A<br>GA	GTCGCGATGCGTCCTCCAGATC
	gadd45	CAACTTCTCAGGCACAG	TGCAGACTCATAAACGC

	eme1	GCTATTGCAAGAGGCAAAGCA	ACATTTTGAATTCTGGGATCTGAAG
	foxm1	TCTCCTGATGGCAAGATCTCCT	TCTGCTGTTGATGGATAGCAACT
	rad9a	GCAGTCCAGTCATCCTGAATGT	TGCATCAGGGCGTTTTGTGT
	rad17	CCAAAATGACCAGGTTTTGCAT	ACCAAATGATGGCTCCACCC
	smc5	CCAAGCTGGACAAGATAAAGACC	GCAACAAGCTGAAAGCTGCAT
	mcm8	TCAGGTGTTGACACTGGATCT	GGAGTCAGTGGCTCATAGTTGT
	blm	GGATTAAAAGGAGCTGATGACGC	ATCCTTTTCCTGTGTGCTCTCT
	mms22l	TGCTGCAAAATGGAGAACTGG	TCTTGTATCTGGAGCATCTGGG
	dtl	TGCACATTTTCCTCCGCTCAT	CATTATCATGTGCCTGCCATTCT
	slx4	GATAAAACGGTGTACATTTCAAAG C	CCTGAATCGTGAACCATGTTG
	tonsl	AAAGAGGAGTGTGAGACGTGG	TAGTCCTGATTTCTCAGCGCA
	ints7	TCACCCTCAGAATGCTGGGAA	TGCTGCAAAATCTTTGGAGTGT
	fanci	GGCTCATCTGCTGTGCTGTA	CTCAGCACACTTATCCAACTCA
	ret	CTATCTGGATCTTGCGGCCT	TGACATGCCATAGAGCTTGTTT
	pax2a	TCTCGGAAGCTATCAAAAGGATCT	CCCAGGCGAACATTGTAGGA
	col11a2	CACTCACTGAACAGGACGTAGA	CGACCTGGTTAATGCTTTCGT
	masp1	CATCGAGGAACTGGCTGTTTTT	GCATTCATCTACATCAATCGCACT
	smad5	CTGTTTGACGCCAGGAGGG	CAAAGTCCACCTCATTCACACA
	spns2	CTGGAAGCATGAGTCCGGC	AAGACTGTTTGCAGCAAGCCC
	kptn	ACCAGTTGATGCAGAAATCGTC	GCTTTGTGCAATAGACTCCAGG
	jag1b	CCGAGGTCCTACACGTTGAT	ATCATGCCAGAGTGGTACGC
	cep290	GCTCAGAAGTTTGAGGAAGCC	TCTTCCAGAATTTTGCAGGTAGT
	wnt11a	CCAGACCTGGAACGAGGAAC	ATGAGCTTGTTGGCGTGAGA
	pi4kβ	CCACCAACGTTCCAAATCCG	GTCAGACGCACAGGCTCATC
	arf1	GGAGGAAACTTGGAACTTGGTG	TGGTGGTCACAATTTCCCCC
	dfna5	TTGGGGAGCACACTAGAGGA	ACTGATGAGCAGGTGTGTGG
	sox9b	CAGCCCAGACGGAGGAAATC	CCCTGAGACTGACCGGAGTG
	pink1	TCGGGAAAGGTTGTAACGCA	ACTTGATGAACCAGCCCCAA
	lmo4a	CGTACAAATAACGGCAGGCG	GATATCCGACCACCGCATCC
	gpr126	GCGCTCACCTGTGATACTGT	ACAGTTAATGTTGCTGGGCA
	ugdh	ATAGGCGCTGGGTATGTTGG	TTCATTCAGGCCCGGCTCAT
	has3	CTGTGTGGTGTGGAAAGGGA	AGTGTCTGAATCACATACCTGC
	rnaseh	GCAGAATTACAGGCTGCTTGC	TTTACCCAACTTGTGACACCAT
Semi-Quan Titativ e PCR	foxm1	GAAGCAAACCGCTGTCTCAC	GTGCCACCCAATGCAGGTAT
	eme1	CATCCAAGTTCCAGTGGATGAC	GTCCCACATTCTTCTCCTCGC
	rad9a	TTGCAGAGTCATCTTGTCTTCCA	AGGAAGTTCGCTGGTCTCCA
	tinf2	TTCAAGTCCAACGCCGGAGAA	CACAGTGCCTGCTCTCTCATC
	rnaseh	AATGTGGCTGAGAGGCTTCC	CCAGCCATTAGACTTCCAGGT
	pold3	GGAAGTGAAAACAGAGCAGAAAG	TTCCAGAAGCCGATGCTGTC
	mcm8	CTAGACTGCTTGGGTGTAGCA	ACTCACTCTCACCACAGTACCT
	baxa	GTGGCAATGACCAGATACTTGAC	ACTCCGGGTCACTTCAGCAT
	tonsl	CCGTGGAGCATTACAGACAAGA	TCTCCTCCTGACAAACAGCCA

	mms22l	CTTTGGCTGGGATGAGATGC	CACACAAAGCTTGGGCTGAAC
	smc5	ACATGACTCCAGGAGAGACGG	CTGTGAAACACTCAGCTCTGG
	usp28(E21-23)	GCATCATGAGAGAGGCCAGA	GGTGTGCGTGTGCCAGATAA
	usp28(E14-15)	AGCCAGAACCAACTCAAGAAC	TCGGGTTGAGAGGACTGACT
	ints7	TGGATGAGTTTGTCAAAAGAGTGT	TCTTGTTGCAAATTCCTGCTGC
	pax2a	GCCATGGCTGTGTCAGCA	ACATTGTAGGATTTTGCCGCTT
	col11a2	CTCCTCTGCCCAAAGTCCAG	CTCGCACTGAGGCACCC
	spns2	ATGAGGTGCACACGCTGTC	TTTGGTAATGAAAGAGCTTAGCAGC
	masp1	AGGTATATTCAGACATCGAGGAAC	TGTTGTCGGAATGAAGCAGG
	eya4	CCCAGTAGACAACAGGGCTT	GCTGGACACTTACACTGGGT
	otog	ATGAAGATTGGGGGCACCAT	TTGCTACGGCAGTCGTTCTT
	cep290	GCTGGTGCAGCAGAATAACC	CTCGAGATAAAACATTCTGTTCTGA
	jag1b	TCAATCGCGAGTTTCCTCGG	GAACTTGTTGCACCCGAAGC
	taz	ATGGTGCACAACCAGGACG	AGGTGTCCATCTCATCTTGTTAAGG
	fgfr2	TTCTGGGTGCTCTCCTGCTA	ATATGGCAGCCGCATTTGTTC
	smad5	AGGGTGAGAGACGAGGGAAA	GGGCAATTGAGGAATCAAGGC
	kptn	AGAAGAAGATCAGACCTGTGGC	GCTGGAAAGGAGTGAACTGGA
	thrb(E7-9)	TGGCATGGCTACAGACTTGG	GGTGCATTGACAATAGGTGCC
	thrb(4-6)	ATACGGGTTGTCACAGAGGC	CTCACAAGGCAGCTCACAGA
	ret	GTGAAAAGCAGAGACTATCTGGA	CTGGCCAGTTCGGGTATGAC
	met	TGAATACGAGGTTCCGGAGAGA	GGATGACGCGGCCTTCA
	GAPDH	GGGGAGCCAAAAGGGTCA	CAGCGTCAAAGGTGGAGGAGT
Genotype Identification	*dhx38*	ACACTGAATGACCTTTGTAAT	TCTCCATCCTCCTGATTTTC
